# Clinical Performance and Survival of Adhesive Attachments for Removable Partial Dentures: A Systematic Review

**DOI:** 10.3390/dj14030174

**Published:** 2026-03-17

**Authors:** Silwan Mendes, Florence Auderset, Nicola Ursula Zitzmann

**Affiliations:** Department of Reconstructive Dentistry, UZB University Center for Dental Medicine Basel, University of Basel, 4058 Basel, Switzerland; florence.auderset@unibas.ch (F.A.); n.zitzmann@unibas.ch (N.U.Z.)

**Keywords:** removable partial dentures, dental attachment, resin-bonded prostheses, prosthesis failure, treatment outcome

## Abstract

**Objectives**: This systematic review evaluated the clinical performance and survival of adhesive attachments used as retention elements for removable partial dentures (RPDs) and analyzed associated biological and technical complications. **Methods**: A systematic electronic search was conducted in PubMed, the Cochrane Library, and Embase in August 2025. Eligible studies included randomized controlled trials, prospective or retrospective clinical studies, and case series with at least 10 patients and a minimum 6-month follow-up. The primary outcome was attachment survival; secondary outcomes included biological and technical complications. Two reviewers independently performed screening, data extraction, and risk of bias assessment using the Newcastle–Ottawa Scale. Due to high heterogeneity, results were analyzed descriptively. This review was prospectively registered (PROSPERO registration number CRD420251116027) and conducted in accordance with PRISMA guidelines. **Results**: Of 5514 records identified, five longitudinal clinical studies fulfilled the inclusion criteria. Sample sizes ranged from 10 to 123 patients, with follow-up periods between 3 and 270 months. Reported attachment survival ranged from 96% at one year to 61% at 15 years. Technical complications, mainly debonding, occurred in 9% to 18.5% of cases, while biological complications such as caries or abutment fractures were infrequent. All studies were rated as poor-quality owing to missing control groups and incomplete outcome reporting. **Conclusions**: Within the limitations of the available evidence, adhesive attachments represent a potential option as an invisible retention element for removable prostheses. However, the available findings are based on a limited number of studies with methodological limitations and therefore represent low-certainty evidence. While some studies suggest favorable short-term performance, long-term survival appears to be limited. Debonding was the most frequently reported technical complication, highlighting the technique sensitivity of adhesive cementation. Further well-designed comparative clinical studies with larger sample sizes are required to better clarify their long-term efficacy and clinical indications within removable prosthodontics.

## 1. Introduction

Partial edentulism remains a highly prevalent condition worldwide, particularly in aging populations, and often requires prosthetic rehabilitation to restore function, esthetics, and oral health-related quality of life [1,2]. Removable dental prostheses, including removable partial dentures (RPDs), continue to play a central role in the treatment of partially edentulous patients, especially when implant treatment and fixed dental prostheses are not feasible due to anatomical, financial, or medical limitations [3]. The prognosis of RPDs largely depends on the effectiveness and durability of their retention elements, which provide stability, distribute occlusal loads, and help to preserve abutment teeth [4].

Conventional retention systems such as cast clasp-retainers, post copings, and telescopic crowns are well established and supported by extensive long-term data. Multiple longitudinal studies have demonstrated survival rates of around 90–96% at five years [5,6,7] and close to 90% at ten years [8,9]. In contrast, Brandt et al. reported lower survival rates of 76.2% after five years and 49.5% after ten years in an investigation of 842 clasp-retained dentures [10]. Despite this strong evidence base, these methods present certain drawbacks, including the esthetic limitations of visible clasps, the invasiveness of telescopic crowns, and an increased risk of technical complications, which in turn may increase the need for maintenance and repair [7,10,11]. In addition, recent evidence indicates that abutment teeth, particularly those that are non-vital, have a significantly increased risk of extraction when used for RPD retention, highlighting the biological challenges associated with the conventional concepts [7,12].

**Figure 1:**
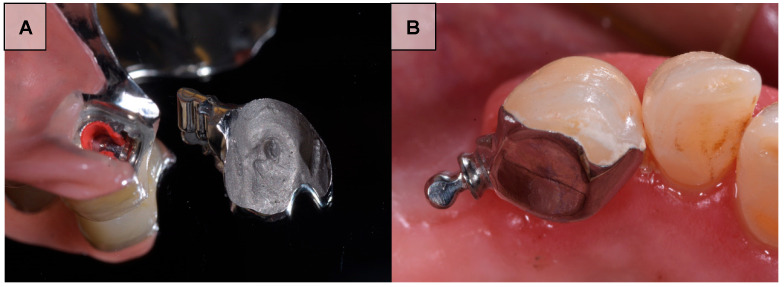
Clinical examples of extracoronal adhesive attachments used for retention of RDP. (**A**) Matrix component incorporated into the RDP engaging the adhesive attachment. (**B**) Adhesive attachment cemented onto the abutment tooth.

To overcome such disadvantages, adhesive attachments—also referred to as resin-bonded attachments—with extracoronal precision components were introduced as a minimally invasive and esthetic alternative for RPD retention. These retention elements are waxed directly onto an investment model and consist of a lingual coverage retainer and an extracoronal patrix, which is provided as a prefabricated castable burn-out resin component [13,14,15]. Castable plastic extracoronal attachments are traditionally classified as semi-precision attachments, since the definitive metal component is fabricated by casting in the dental laboratory. However, because both the plastic patterns of the patrix and the corresponding matrices are industrially prefabricated with high precision, such attachments may clinically be considered precision attachments. With contemporary digital workflows, the design can be performed entirely in the digital environment. The extracoronal attachment is selected from the software library, positioned according to the desired path of insertion, and subsequently milled in burnout resin. After casting in a CoCrMo alloy, the adhesive attachments are bonded to the enamel surface of the abutment tooth using resin cement, while an interchangeable acrylic resin insert is incorporated into the RPD as the matrix component. Clinical examples of adhesive attachments used for RPD retention are shown in Figure 1. The proposed advantages include preservation of tooth structure, simplified preparation, reduced treatment costs, and improved esthetics [16]. However, adhesive attachments are inherently technique-sensitive, and clinical success depends on adequate abutment preparation with rests and guiding grooves, effective conditioning of non-precious alloy surfaces, and meticulous adhesive cementation under strict moisture control [17,18,19].

Against this background, the present systematic review aims to evaluate the survival of adhesive attachments used as retention elements in removable partial dentures (RPDs) and to document associated biological and technical complications. By synthesizing the available clinical evidence, this review seeks to provide an overview of the reported clinical performance of adhesive attachments and their potential role in contemporary removable prosthodontics.

## 2. Materials and Methods

**Figure 2:**
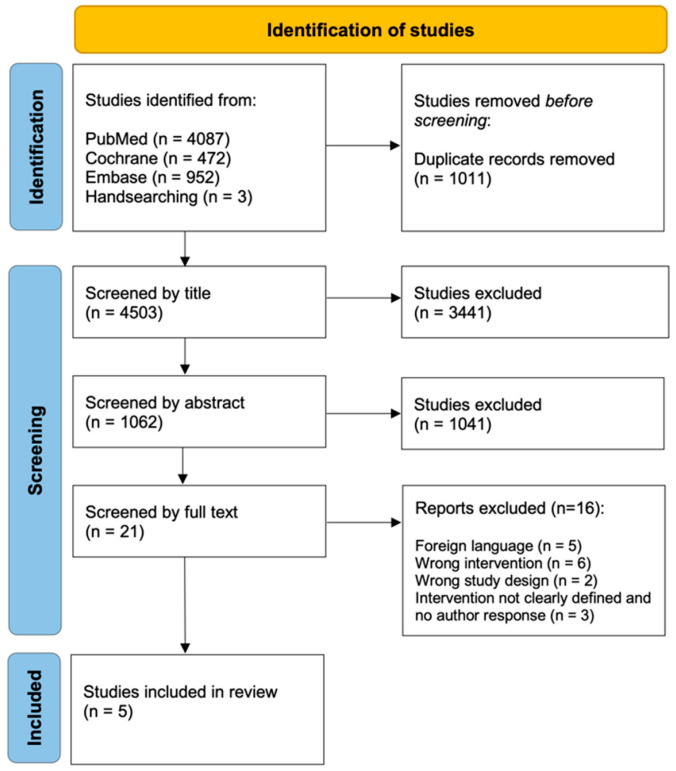
PRISMA flowchart illustrating the selection process of studies included in the systematic review and the reasons for exclusion.

This review was prospectively registered in the International Prospective Register of Systematic Reviews (PROSPERO, registration number CRD420251116027) prior to the start of data screening. It was conducted in accordance with the PRISMA (Preferred Reporting Items for Systematic Reviews and Meta-Analyses) guidelines [20]. The completed PRISMA 2020 checklist is provided in Supplementary Table S1. A PRISMA flowchart was created to illustrate the study selection process (Figure 2). The present systematic review followed key methodological principles recommended by the Cochrane Collaboration [21], including a structured PICO approach, dual independent screening, PRISMA documentation, and formal risk of bias assessment.

The focused research question “Are adhesive attachments a successful and effective therapy for retention of RPD in partially edentulous patients?” was analyzed using the PICO framework:

- Population (P): Partially edentulous patients rehabilitated with Removable dental prostheses.
- Intervention (I): Use of adhesive attachments as retention elements.
- Comparison (C): Conventional retention systems including cast metal frameworks with clasp retention, telescopic crowns, crowns with intra- or extracoronal attachments, and/or single-implant-supported elements.
- Outcome (O): The primary outcome was the survival of the adhesive attachment. Secondary outcomes considered biological and technical complications for success rates, patient satisfaction and quality of life.

### 2.1. Eligibility Criteria

Studies were eligible if they met the following criteria:

- A minimum of 10 patients included
- A minimum follow-up period of 6 months
- Study types: Randomized controlled trials, prospective or retrospective clinical studies, and case series with at least 10 cases
- Articles published in English or German

Exclusion criteria were studies on ceramic adhesive attachments and studies involving patients with cleft lip and palate.

### 2.2. Search Strategy

A systematic electronic search was conducted in PubMed on 19 August 2025, using the following strategy:

(“removable partial denture*” OR “removable dental prosthesis*” OR “removable dental prostheses*” OR “partial denture*” OR “removable prosthesis*” OR “overdenture*”) AND (“attachment*” OR “adhesive attachment*” OR “adhesive retainer*” OR “locator*” OR “ball attachment*” OR “bar attachment*” OR “stud attachment*” OR “telescope attachment*” OR “opa attachment*” OR “ceka attachment*” OR “swing-lock” OR “precision attachment*” OR “friction attachment*” OR “retention element*” OR “retention system*” OR “resin bonded” OR “resin-bonded”).

To maximize the sensitivity of the search strategy, terms commonly associated with implant-supported removable prostheses, such as locator, stud attachment, and bar attachment, were also included, as they are classified under the broader category of attachments in the Glossary of Prosthodontic Terms. Their inclusion aimed to ensure that potentially relevant studies related to adhesive attachments were not inadvertently excluded from the literature search. Title searches were conducted in the Cochrane Library and Embase.

Duplicate records were removed. Two independent reviewers (SM and FA) screened titles, abstracts, and full texts. Disagreements were resolved through discussion and consensus. In case of unclear or missing information, study authors were contacted directly. Additional relevant studies were identified through hand-searching the reference lists of included and relevant studies.

### 2.3. Data Extraction and Analysis

Data extraction was performed independently by two reviewers (SM and FA) using a structured Excel spreadsheet. Extracted variables included study design, sample size, number of adhesive attachments, patient age, abutment tooth characteristics, attachment type, intervention details, comparison group, follow-up duration, outcomes (attachment survival, biological and technical complications affecting attachment success), and conflict of interest.

### 2.4. Risk of Bias Assessment

The methodological quality of the included studies was assessed independently by two reviewers (SM and FA) using the Newcastle–Ottawa Scale (NOS) [22], which was considered appropriate as no randomized controlled trials were found. As the NOS provides no predefined thresholds, cut-offs commonly used in the literature were adopted, classifying studies as good (7–9 points), fair (4–6 points), or poor (0–3 points). According to this scale, studies without a comparison group received 0 points in the comparability domain, which automatically resulted in a classification as poor quality [23].

### 2.5. Data Preparation

Extracted data were tabulated in a standardized format. For one study [15], the distribution of follow-up durations was estimated based on reported mean, standard deviation (SD), and range, assuming a normal distribution to provide an approximate proportion of short-term cases. This estimate was used only for descriptive purposes. No further data conversions, imputations, or statistical transformations were performed.

The included studies showed substantial variability in study design, intervention protocols, outcome measures, and follow-up durations, which precluded statistical pooling of data. Due to considerable heterogeneity across studies, no quantitative meta-analysis was conducted. Instead, results were summarized descriptively in comparative tables.

## 3. Results

### 3.1. Study Selection

The search yielded 5514 records. After removal of 1011 duplicates, 4503 titles were screened. A total of 1062 abstracts were reviewed, of which 21 full texts were assessed. Five studies ultimately met the inclusion criteria. Exclusions were due to foreign language, wrong intervention, inappropriate study design, or undefined intervention. Authors of studies with ambiguously defined interventions were contacted by email, but no responses were received, so these studies were excluded. Details of the study selection process are presented in the PRISMA flowchart (Figure 2).

### 3.2. Study Characteristics

The five included studies were all longitudinal in design, four were published between 1987 and 1997 and one in 2024. All studies were conducted at university dental schools, i.e., two research groups in Switzerland (3 studies), one in Germany, and one in Austria. Sample sizes ranged from 10 to 123 patients. The number of prostheses was indicated in three studies, which documented 12, 17, and 18 prostheses, respectively [14,24,25]. Follow-up times ranged from 3 to 270.6 months with mean follow-ups between 12 (no SD indicated) and 84.5 ± 51.3 months, while one study reported a fixed observation period of 48 months (Table 1) [26].

### 3.3. Intervention

All studies defined inclusion criteria for abutment teeth, most often referring to enamel integrity, periodontal conditions, and available interocclusal space (Table 1). The single adhesive attachments used were Ceka Preci-Line, Roach, SG, Biloc, and Regulex. Surface pretreatments such as aluminum oxide air-abrasion, etching of attachment and tooth, and placement of a rubber dam were consistently reported. The composite cements used for adhesive cementation were Comspan opaque (Caulk Division Dentsply) in three studies, while Superbond (Sun Medical), and Panavia 21 EX or Panavia V5 (Kuraray Noritake) were each used in one study. Only one study [14] included a comparison group of 101 patients treated with resin-bonded fixed dental prostheses, although no direct comparisons were performed (Table 1).

### 3.4. Risk of Bias Within Studies

All five studies were judged to be of poor quality, as the absence of control groups with alternative retainers resulted in 0 points in the Comparability domain of the Newcastle–Ottawa Scale. Additional limitations included insufficient outcome assessment and short follow-up durations (Table 2). No meta-analysis was performed because the available data were too heterogeneous to allow for statistical pooling of results. An overlap of data in the two studies by Marinello & Schärer, 1987, and Marinello & Schärer, 1990, could not be ruled out.

### 3.5. Primary Outcome – Survival of Adhesive Attachments

In three studies, the reference periods for the reported survival rates were unclear [14,24,25]. The survival of adhesive attachments in these studies ranged from 91% at 12 months to 100% at 14 months [14,24,25]. Marinello & Schärer (1990) reported that the survival rate decreased from 96% at one year to 80% after four years [26]. Garling et al. (2024) presented the longest follow-up, with survival of 68.3% at 10 years and 61% at 15 years [15].

### 3.6. Secondary Outcomes

#### 3.6.1. Biological Complications

In the early studies, tooth survival was high, with few biological complications. Marinello & Schärer (1987), Besimo et al. (1997), and Schäffer et al. (1990) reported no biological failures at all. Marinello & Schärer (1990) reported only a single biological complication with a fractured abutment tooth (2%). The most recent study by Garling et al. (2024) observed a higher incidence over longer follow-up, with abutment fractures being the most frequent complication (7.3%), alongside cases of caries (3.4%) and periodontal disease (2.9%).

#### 3.6.2. Technical Complications

Technical complications occurred in 8.3% to 18.5% of cases [14,15]. Marinello & Schärer (1990) reported an overall success rate of 86% after two years, with debonding as the primary complication, which occurred only in non-prepared abutment teeth. Garling et al. (2024) reported markedly lower long-term outcomes, with success rates of 58.4% at 10 years and 46.2% at 15 years, again with debonding as the most common technical complication. Only one study [25] recorded a 100% success rate, although the authors noted that matrices required activation every six months for Ceka attachments and Dolder bars, and four prostheses required relining.

Patient satisfaction and quality of life were not addressed in any of the included studies.

## 4. Discussion

This systematic review focused on the survival of adhesive attachments used for retention of removable dental prostheses and documented very limited evidence, as most of the included studies were published decades ago, involved small patient groups with short follow-up periods, and often reported outcomes inconsistently. When compared with conventional retention elements such as clasps, telescopes, or post copings [5,6,7], adhesive attachments showed comparable short-term outcomes, with survival rates ranging from 91% to 100% after 1 to 4 years. The most recent study included reported long-term data showing that the survival rate of adhesive attachments decreased to 68% after 10 years [15].

Debonding was consistently the most frequent complication, highlighting the technique-sensitive nature of adhesive systems. These failures were therefore predominantly technical and may be associated with degradation of the adhesive interface over time, occlusal loading, or insufficient bonding protocols. These long-term data are within the range of survival of clasp-retained RPDs, which was 76.2% after 5 years and 49.5% after 10 years in a study including 842 RPDs [10], while 89.9% survival was reported after 10 years despite the high rate of fractures in clasps, major or minor connectors affecting 16.1%, 5.1% and 3.4% of the 174 RPDs [8].

In addition to the technical challenges, which require adequate retentive preparation and proper pretreatment of enamel and non-precious alloys, biological complications such as secondary caries and abutment fractures also affected the long-term prognosis of adhesive attachments. Overall, the available evidence suggests that long-term failures were mainly technical (debonding), while biological complications were reported less frequently. These findings emphasize that clinical outcomes strongly depend on procedural accuracy, as even minor deviations may compromise long-term survival. Consequently, patients should be informed about the possible risks of complications and the necessity for regular recall visits, which are essential to detect and address problems at an early stage [24]. Given these limitations, adhesive attachments should be used cautiously and only under clear indications.

However, the minimally invasive concept of adhesive attachments also demonstrated potential benefits compared with conventional retention systems. In the present review, failures from biological complications were reported in only two of the included studies with a single case (2%) of abutment fracture after 3 years [26], and 7.3% fractures documented in the most recent study after a mean observation period of 7 years [15]. The latter also reported biological complications with caries (3.4%) and periodontal disease (2.9%), which affected success but not necessarily survival [15]. Biological complications such as caries and periodontal disease were found in 17.9% of clasp-retained RPDs resulting in loss of abutment teeth after a mean observation time of 3.5 years in a study including 842 RPDs [10]. In a recent study investigating the more invasive concept of telescopic crowns, root fractures affected 15% of abutments after 3.9 years, and an additional 10% of fractured core build-up crown complexes were classified as technical complications [7]. Root fractures were also the main cause of post coping failures, affecting 7.9% of 152 post copings after nearly 9 years [5].

None of the studies included in this review evaluated oral health–related quality of life (OHRQoL), and to date there are no data available on OHRQoL for extracoronal adhesive attachments. This represents an important gap in the current evidence, as patient-reported outcomes such as quality of life and treatment satisfaction are central to the clinical rationale for minimally invasive retention concepts. Two investigations on crowns with extracoronal precision attachments reported improved OHRQoL compared to clasp-retained RPDs [27,28], suggesting potential patient-centered benefits that remain to be confirmed for RPD retained by adhesive attachments.

This review followed a systematic methodology with a clearly defined PICO question and a comprehensive search strategy. It is one of the few systematic attempts to address the question of adhesive attachments in removable prosthodontics. Nevertheless, the review is limited by the small number of eligible studies, most of which were old and methodologically weak. Given the considerable evolution of adhesive materials, surface treatment protocols, and bonding strategies over the past decades, the applicability of these historical data to current clinical practice may be limited. In addition, several of the included studies were case series with small sample sizes, increasing the risk of selection and reporting bias and further limiting the strength of the conclusions. There may also have been overlap in patient populations between two studies [24,26], which could have duplicated data in the analysis. Publication bias cannot be excluded, as language restrictions were applied and negative results may remain unpublished. Furthermore, unanswered author inquiries contributed to incomplete data retrieval.

The evidence base for adhesive attachments is characterized by high heterogeneity. Differences in study design, patient populations, types of attachment systems, and outcome measures make direct comparisons difficult. None of the included studies incorporated control groups with alternative retention elements, and definitions of success and survival varied widely, further complicating interpretation. Consequently, the reported survival rates should be interpreted as study-specific findings and are not directly comparable across studies. Moreover, the lack of standardized protocols for follow-up and outcome reporting reduced the ability to synthesize data across studies. Overall, the methodological quality of the included studies was poor in the risk of bias assessment, and the inclusion of case series alongside cohort studies further limits the internal validity and generalizability of the findings, limiting the strength of the conclusions. Consequently, the available evidence does not allow for firm clinical recommendations regarding the use of adhesive attachments in removable prosthodontics.

Future research should focus on prospective, randomized, and long-term clinical trials with larger sample sizes and direct comparison groups. Standardized definitions of success and survival are needed to enable comparability across studies. Importantly, patient-centered outcomes such as quality of life, satisfaction, and cost-effectiveness should be integrated as primary endpoints, as they are currently underrepresented. The true long-term clinical relevance of adhesive attachments can only be determined through well-designed and adequately powered studies.

## 5. Conclusions

Within the limitations of the available evidence, adhesive attachments for removable partial dentures (RPDs) may show favorable short-term performance, although a tendency toward reduced long-term survival has been reported. However, these findings are based on a limited number of studies with methodological limitations and therefore represent low-certainty evidence. Debonding was the most frequent complication, reflecting the technique sensitivity of adhesive procedures and the need for meticulous adhesive cementation. Biological complications, including caries and abutment fractures, were rarely reported in the included studies. While adhesive attachments offer a minimally invasive and esthetic alternative to conventional retention systems, their long-term efficacy remains uncertain. Therefore, no firm clinical recommendations can currently be drawn based on the available evidence. Further high-quality comparative studies are required to clarify their role in removable prosthodontics.

## Figures and Tables

**Table 1:** Characteristics of included studies.

Author, Year, Origin	Study Design	Sample Size (Patients, RPDs)	Number of Single Adhesive Attachments/Number of Other Attachments	Age, Mean ± SD (Range) in years	Abutment Tooth Characteristics	Attachment Type	Intervention Details	Comparison Group	Follow-Up Duration, Mean (Range)	Primary Outcome (Survival)	Secondary Outcomes (Success)	Conflict of Interest
>Marinello & Schärer 1987 [24], Zurich Switzerland	longitudinal	*n* = 15 patients (5 women, 10 men) *n* = 17 RPDs (3 max, 14 mand)	30/4	n.a.	sufficient enamel (unrestored and nondecayed) on lingual and proximal surfaces, mounted study casts, evaluation of the interocclusal space	Biloc attachment (*n* = 11), Roach attachment (*n* = 17), Regulex attachment (*n* = 2), Dolder bar (*n* = 2), Cantilever pontic (*n* = 2)	retentive abutment preparation in 11 abutments (pilot-phase with no preparation in 23 abutments), cleaning of the adhesive framework with 50 µm aluminum oxide abrasive, electrochemical etching and cleaning with 18% hydrochloric acid, etching enamel surface with 37% phosphoric acid gel for 60 s, cementation with Comspan opaque under isolation with rubber dam	no comparison group	12 mo (3.5–27.7 mo)	91% ^a^	91% success ^a^ -biological complications: 0% -technical complications: 9% (debonding of the attachment after 5.5 mo in 1 patient, and after 16.2 mo and 21.4 mo in another patient) all in non-prepared abutments	not mentioned
Schäffer et al., 1990 [25], Innsbruck Austria	longitudinal	*n* = 16 *n* = 18 RPDs (4 max, 14 mand)	9/29	n.a.	periodontally healthy, free of caries, adequate enamel surface, excellent oral hygiene, periapical radiograph of the abutment tooth available, clinical and articulator-based functional analysis	Roach attachment (*n* = 7) ^b^, Ceka attachment or Dolder bar (*n* = 29)	retentive abutment preparation; electrochemical etching of the adhesive framework, cementation with Superbond, etching enamel surface with 37% phosphoric acid gel for 60 s	no comparison group	14 mo (3–52 mo)	100% ^a^	100% success ^a^ -biological complications: 0% -technical complications: activation of matrices every 6 mo on average (only Ceka and Dolder bar), relining of 4 mandibular RPDs (attachment type not indicated)	not mentioned
Marinello & Schärer, 1990 [26], Zurich Switzerland	longitudinal	*n* = 26	50/0	25–83 Mean57)	free of caries, no fillings, positive sensitivity testing of all abutment teeth except for one, articulator-based functional analysis	Roach attachment	pilot-phase with no preparation, afterwards retentive abutment preparation (number not indicated), electrochemical etching of adhesive framework, cementation with Comspan opaque under isolation with rubber dam	no comparison group	48 mo	96% after 1 yr 85% after 2 yr 80% after 30 mo up to 48 mo	86% success after 2 yr -biological complications: 2% (fracture of one abutment tooth, which was the only non-vital abutment) -technical complications: 12% (5cases debonding all in non-prepared abutments, one case removal of attachment in splinted situation) ^c^	not mentioned, potential overlap with Marinello et al. 1987 [24]
Besimo et al., 1997 [14], Basel Switzerland	longitudinal	*n* = 10 patients (4 women, 6 men) *n* = 12 RPDs (3 max, 9 mand) comparison *n* = 101	24/0	36–70 (∅54)	positive sensitivity testing of all abutments, no fillings on the surfaces used for bonding, articulator-based functional analysis	SG attachment	retentive abutment preparation, electrolytic etching, cementation with Comspan opaque, etching enamel surface with 37% orthophosphoric acid, using rubber dam for placing the restoration	resin-bonded fixed dental prostheses (RBFDP)	Mean 28 months (4–56 mo)	91.7% ^a^	91.7% success ^a^ -biological complications: 0% -technical complications: 8.3% (2 cases debonding of the attachment after 4 mo in 2 patients) ^d^ RBFDP 97% at 3 yr, 95% at 5 yrs -biological complications: 0% -technical complications: 4.7% (6 cases debonding of one retainer)	not mentioned
Garling et al., 2024 [15], Kiel Germany	longitudinal	*n* = 123 (62 women, 61 men)	205/0	36–83 (∅63.6 ± 9.6)	free of caries and fillings (or minor defects that did not affect the planned retainer surface), bonding area solely on enamel at least 30 mm ^2^, periodontally healthy, appropriate occlusion that allows for the application of a retainer wing with a thickness of at least 0.5 mm	Ceka Preci-Line attachment	retentive abutment preparation, airbrushing of the bonding surface with 50 µm aluminum oxide abrasive (2.5 bar pressure) and ultrasonically cleaning in 99% isopropanol, etching enamel surface with phosphoric acid, cementation with Panavia 21 EX or Panavia V5 under isolation with rubber dam	no comparison group	Mean 84.5 ± 51.3 mo (3.6–270.6 mo)	68.3% after 10 yr 61% after 15 yr	58.4% success after 10 yr 46.2% success after 15 yr -biological complications: 13.6% (7 cases caries, 6 cases periodontal disease, 15 cases fracture of the abutment tooth) -technical complications: 18.5% (33 cases debonding, 5 cases fracture of the alloy cast)	not mentioned

n.a. = not available; mand = mandibular; max = maxillary; mo = months; RPD = removable partial denture; RBFDP = resin-bonded fixed dental prosthesis; yr = years. ^a^ Reference period for success and survival rates not clearly indicated. ^b^ Roach attachments were preferred for single abutments (n = 7); no details for two remaining cases. ^c^ Debonded attachments were recemented. ^d^ Debonded attachments were replaced by conventional metal-ceramic crowns with extracoronal attachments.

**Table 2:** Risk of bias assessment of included studies using the Newcastle–Ottawa Scale (NOS).

Studies	Selection				Comparability	Outcome			Total Quality Score
Author, Year	Representativeness of the Exposed Cohort	Selection of the Non-Exposed Cohort	Ascertainment of Exposure	Demonstration That Outcome of Interest Was Not Present at Start of Study	Comparability of Cohorts Based on the Design or Analysis	Assessment of Outcome	Was Follow-Up Long Enough for Outcomes to Occur	Adequacy of Follow-Up of Cohorts	
Marinello & Schärer, 1987 [24]	1	0	1	1	0	1	0 *	1	6 poor quality
Schäffer et al., 1990 [25]	1	0	1	1	0	1	0 *	1	6 poor quality
Marinello & Schärer, 1990 [26]	1	0	1	1	0	1	1	1	7 poor quality
Besimo et al., 1997 [14]	1	0	1	1	0 **	1	0 *	1	6 poor quality
Garling et al., 2024 [15]	1	0	1	1	0	1	1 ***	1	7 poor quality

* Mean follow-up was 12–28 months with a minimum of only 3 respectively 4 months. No distribution could be estimated due to missing standard deviations. ** Besimo et al. included a comparison group, but no direct comparison of data was performed; therefore, 0 points were assigned in the comparability domain. *** Mean follow-up was 84.5 ± 51.3 months with a range of 3.6–270.6 months. Assuming a normal distribution, approximately 10% of cases fell between 3.6 and 33 months; however as follow-up times are typically right-skewed, this estimate should be interpreted with caution. Note: Commonly applied cut-offs were used to classify studies as good (7–9 points), fair (4–6 points), or poor (0–3 points). Studies lacking a comparison group automatically scored 0 in the comparability domain, resulting in a poor-quality rating.

## Data Availability

All data analyzed in this study are derived from previously published studies, which are all publicly available and listed in the reference list.

## Supplementary Materials

The following are available online at https://www.mdpi.com/article/10.3390/dj14030174/s1, Table S1: PRISMA 2020 Checklist.

## Abbreviations

The following abbreviation is used in this manuscript:

RPD	Removable Partial Dentures

## References

[1] Al-Rafee M.A. (2020). The epidemiology of edentulism and the associated factors: A literature Review. J. Fam. Med. Prim. Care.

[2] Kassebaum N.J., Bernabé E., Dahiya M., Bhandari B., Murray C.J.L., Marcenes W. (2013). Global burden of oral conditions in 1990–2010: A systematic analysis. J. Dent. Res..

[3] Campbell S.D., Cooper L., Craddock H., Hyde T.P., Nattress B., Pavitt S.H., Seymour D.W. (2017). Removable partial dentures: The clinical need for innovation. J. Prosthet. Dent..

[4] Wöstmann B., Budtz-Jørgensen E., Jepson N., Mushimoto E., Palmqvist S., Sofou A., Owall B. (2005). Indications for removable partial dentures: A literature review. Int. J. Prosthodont..

[5] Kraljevic I., Glenz F., Jordi C., Zimmermann S.D., Joda T., Zitzmann N.U. (2020). Long-term observation of post copings retaining overdenture prostheses. Int. J. Prosthodont..

[6] Rehmann P., Wöstmann B., Orbach K., Ferger P. (2013). Treatment outcomes with removable partial dentures: A retrospective analysis. Int. J. Prosthodont..

[7] Klotz A.-L., Hagspiel S., Büsch C., Zenthöfer S., Rammelsberg P., Zenthöfer A. (2024). Mid-term survival and complications of double-crown-retained removable dental prostheses placed in the dental practice—A retrospective study. Clin. Oral. Investig..

[8] Behr M., Zeman F., Passauer T., Koller M., Hahnel S., Buergers R., Lang R., Handel G., Kolbeck C. (2012). Clinical performance of cast clasp–retained removable partial dentures: A retrospective study. Int. J. Prosthodont..

[9] Kern J.-S., Hanisch O., Hammächer C., Yildirim M., Wolfart S. (2019). Telescopic crowns on implants and teeth: Evaluation of a clinical study after 8 to 12 years. Int. J. Oral. Maxillofac. Implant..

[10] Brandt S., Winter A., Lauer H.-C., Romanos G. (2024). Retrospective clinical study of 842 clasp retained removable partial dentures with a metal framework: Survival, maintenance needs, and biologic findings. Quintessence Int..

[11] Shala K.S., Dula L.J., Pustina-Krasniqi T., Bicaj T., Ahmedi E.F., Lila-Krasniqi Z., Tmava Dragusha A. (2016). Patient’s satisfaction with removable partial dentures: A retrospective case series. Open Dent. J..

[12] Drummond L.B., Bezerra A.P., Feldmann A., Gonçalves T.M.S.V. (2025). Long-term assessment of the periodontal health of removable partial denture wearers: A systematic review and meta-analysis. J. Prosthet. Dent..

[13] Marinello C.P., Schärer P., Meyenberg K. (1991). Resin-bonded etched castings with extracoronal attachments for removable partial dentures. J. Prosthet. Dent..

[14] Besimo C., Gächter M., Jahn M., Hassell T. (1997). Clinical performance of resin-bonded fixed partial dentures and extracoronal attachments for removable prostheses. J. Prosthet. Dent..

[15] Garling A., Krummel A., Kern M. (2024). Outcomes of resin-bonded attachments for removable dental prostheses. J. Prosthodont. Res..

[16] Carneiro C.A., Santiago Junior J.F., Peralta L.C.F., Neppelenbroek K.H., Porto V.C. (2024). What is the best tooth-supported attachment system for distal-extension removable partial dentures? A systematic review with meta-analysis. Int. J. Prosthodont..

[17] Kern M., Thompson V.P. (1995). Durability of resin bonds to a cobalt-chromium alloy. J. Dent..

[18] O’ Connor C., Gavriil D. (2021). Predictable bonding of adhesive indirect restorations: Factors for success. Br. Dent. J..

[19] Belkhode V.M., Nimonkar S.V., Godbole S.R., Nimonkar P., Sathe S., Borle A. (2019). Evaluation of the effect of different surface treatments on the bond strength of non-precious alloy-ceramic interface: An SEM study. J. Dent. Res. Dent. Clin. Dent. Prospect..

[20] Page M.J., McKenzie J.E., Bossuyt P.M., Boutron I., Hoffmann T.C., Mulrow C.D., Shamseer L., Tetzlaff J.M., Akl E.A., Brennan S.E. (2021). The PRISMA 2020 statement: An updated guideline for reporting systematic reviews. BMJ.

[21] Higgins J.P.T., Thomas J., Chandler J., Cumpston M., Li T., Page M.J., Welch V.A. (2019). Cochrane Handbook for Systematic Reviews of Interventions.

[22] Wells G.A., Shea B., O’Connell D., Peterson J., Welch V., Losos M., Tugwell P. (2022). The Newcastle–Ottawa Scale (NOS) for Assessing the Quality of Nonrandomized Studies in Meta-Analyses.

[23] Stang A. (2010). Critical evaluation of the Newcastle-Ottawa scale for the assessment of the quality of nonrandomized studies in meta-analyses. Eur. J. Epidemiol..

[24] Marinello C.P., Schärer P. (1987). Resin-bonded etched cast extracoronal attachments for removable partial dentures: Clinical experiences. Int. J. Periodontics Restor. Dent..

[25] Schäffer H. (1990). Clinical results of partial denture anchorage using extracoronal bonded attachments. Dtsch. Zahnarztl. Z..

[26] Marinello C.P., Schärer P. (1990). Extracoronal adhesive attachments: Innovative, esthetic option in partial dentures. Phillip-J. Zahnärztliche Prax..

[27] Swelem A.A., Abdelnabi M.H. (2021). Attachment-retained removable prostheses: Patient satisfaction and quality of life assessment. J. Prosthet. Dent..

[28] Peršić S., Kranjčić J., Kovačević Pavičić D., Lajner Mikić V., Čelebić A. (2017). Treatment outcomes based on patients’ self-reported measures after receiving new clasp or precision attachment retained removable partial dentures. J. Prosthodont. Off. J. Am. Coll. Prosthodont..

