# Supplementary material for: Clinical Performance and Survival of Adhesive Attachments for Removable Partial Dentures: A Systematic Review
Source: Dent J (Basel). 2026 Mar 17;14(3):174. doi: 10.3390/dj14030174 (PMC13025724; doi:10.3390/dj14030174)
Supplement: Supplementary file 1 [file dentistry-14-00174-s001.zip › dentistry-4125779-supplementary.pdf]

PRISMA 2020 Checklist

Section and Topic	Item #	Checklist item	Location where item is reported
**TITLE**			
Title	1	Identify the report as a systematic review.	Page 1, line 13
**ABSTRACT**			
Abstract	2	See the PRISMA 2020 for Abstracts checklist.	See PRISMA 2020 for Abstracts checklist (attached)
**INTRODUCTION**			
Rationale	3	Describe the rationale for the review in the context of existing knowledge.	See introduction section; page 1-2, lines 41-73
Objectives	4	Provide an explicit statement of the objective(s) or question(s) the review addresses.	See introduction section; page 2, lines 74-80
**METHODS**			
Eligibility criteria	5	Specify the inclusion and exclusion criteria for the review and how studies were grouped for the syntheses.	See methods section; page 3, lines 106-114
Information sources	6	Specify all databases, registers, websites, organisations, reference lists and other sources searched or consulted to identify studies. Specify the date when each source was last searched or consulted.	See methods section; pages 3-4, lines 115-130
Search strategy	7	Present the full search strategies for all databases, registers and websites, including any filters and limits used.	See methods section; pages 3-4, lines 115-130
Selection process	8	Specify the methods used to decide whether a study met the inclusion criteria of the review, including how many reviewers screened each record and each report retrieved, whether they worked independently, and if applicable, details of automation tools used in the process.	See methods section; page 4, lines 126-130
Data collection process	9	Specify the methods used to collect data from reports, including how many reviewers collected data from each report, whether they worked independently, any processes for obtaining or confirming data from study investigators, and if applicable, details of automation tools used in the process.	See methods section; page 4, lines 131-137
Data items	10a	List and define all outcomes for which data were sought. Specify whether all results that were compatible with each outcome domain in each study were sought (e.g. for all measures, time points, analyses), and if not, the methods used to decide which results to collect.	See methods section; page 4, lines 131-137
	10b	List and define all other variables for which data were sought (e.g. participant and intervention characteristics, funding sources). Describe any assumptions made about any missing or unclear information.	See methods section; page 4, lines 131-137
Study risk of bias assessment	11	Specify the methods used to assess risk of bias in the included studies, including details of the tool(s) used, how many reviewers assessed each study and whether they worked independently, and if applicable, details of automation tools used in the process.	See methods section; page 4, lines 138-145
Effect measures	12	Specify for each outcome the effect measure(s) (e.g. risk ratio, mean difference) used in the synthesis or presentation of results.	See table 1. Effect measures were percentages (survival rates and proportions of biological or technical complications) as reported in the original studies.
Synthesis methods	13a	Describe the processes used to decide which studies were eligible for each synthesis (e.g. tabulating the study intervention characteristics and comparing against the planned groups for each synthesis (item #5)).	See table 1. All included studies fulfilled the eligibility criteria and were comparable regarding key characteristics, allowing qualitative synthesis.
	13b	Describe any methods required to prepare the data for presentation or synthesis, such as handling of missing summary statistics, or data conversions.	See methods section; page 4, lines 152-156
	13c	Describe any methods used to tabulate or visually display results of individual studies and syntheses.	See tables 1&2. Study characteristics, outcomes, and risk of bias were summarized in tables and described

Section and Topic	Item #	Checklist item	Location where item is reported
			narratively in the text. No quantitative synthesis or graphical display was performed.
	13d	Describe any methods used to synthesize results and provide a rationale for the choice(s). If meta-analysis was performed, describe the model(s), method(s) to identify the presence and extent of statistical heterogeneity, and software package(s) used	See methods section; page 4, lines 152-156<br>Results were summarized descriptively in text and tables.
	13e	Describe any methods used to explore possible causes of heterogeneity among study results (e.g. subgroup analysis, meta-regression).	No formal assessment of heterogeneity was performed, as only a qualitative synthesis was conducted.
	13f	Describe any sensitivity analyses conducted to assess robustness of the synthesized results.	No sensitivity analysis was performed, as only a qualitative synthesis was conducted.
Reporting bias assessment	14	Describe any methods used to assess risk of bias due to missing results in a synthesis (arising from reporting biases).	No formal assessment of reporting bias was performed, as only a qualitative synthesis without meta-analysis was conducted.
Certainty assessment	15	Describe any methods used to assess certainty (or confidence) in the body of evidence for an outcome.	No formal assessment of certainty was performed, as only a qualitative synthesis of heterogeneous studies was conducted.
**RESULTS**			
Study selection	16a	Describe the results of the search and selection process, from the number of records identified in the search to the number of studies included in the review, ideally using a flow diagram.	See results section; page 4, lines 159-165; see figure 1
	16b	Cite studies that might appear to meet the inclusion criteria, but which were excluded, and explain why they were excluded.	See results section; page 4, lines 162-165
Study characteristics	17	Cite each included study and present its characteristics.	See table 1
Risk of bias in studies	18	Present assessments of risk of bias for each included study.	See table 2
Results of individual studies	19	For all outcomes, present, for each study: (a) summary statistics for each group (where appropriate) and (b) an effect estimate and its precision (e.g. confidence/credible interval), ideally using structured tables or plots.	See table 1
Results of syntheses	20a	For each synthesis, briefly summarise the characteristics and risk of bias among contributing studies.	See results section; page 14, lines 206-231; see tables 1&2
	20b	Present results of all statistical syntheses conducted. If meta-analysis was done, present for each the summary estimate and its precision (e.g. confidence/credible interval) and measures of statistical heterogeneity. If comparing groups, describe the direction of the effect.	See results section; page 14, lines 206-231. No statistical synthesis or meta-analysis was performed. Results were summarized descriptively in text and tables.
	20c	Present results of all investigations of possible causes of heterogeneity among study results.	No investigation of heterogeneity was performed, as only a qualitative synthesis without meta-analysis was conducted.
	20d	Present results of all sensitivity analyses conducted to assess the robustness of the synthesized results.	No sensitivity analysis was conducted, as only a qualitative synthesis without meta-analysis was performed.

PRISMA 2020 Checklist

Section and Topic	Item #	Checklist item	Location where item is reported
Reporting biases	21	Present assessments of risk of bias due to missing results (arising from reporting biases) for each synthesis assessed.	No formal assessment of reporting bias was conducted, as only a qualitative synthesis without meta-analysis was performed.
Certainty of evidence	22	Present assessments of certainty (or confidence) in the body of evidence for each outcome assessed.	No formal assessment of certainty or confidence in the body of evidence was conducted, as only a qualitative synthesis was performed.
**DISCUSSION**			
Discussion	23a	Provide a general interpretation of the results in the context of other evidence.	See discussion section; pages 14-15; lines 233-275
	23b	Discuss any limitations of the evidence included in the review.	See discussion section; page 15; lines 278-292
	23c	Discuss any limitations of the review processes used.	See discussion section; page 15; lines 285-292
	23d	Discuss implications of the results for practice, policy, and future research.	See discussion section; page 15; lines 293-299
**OTHER INFORMATION**			
Registration and protocol	24a	Provide registration information for the review, including register name and registration number, or state that the review was not registered.	See methods section; page 2; lines 82-84
	24b	Indicate where the review protocol can be accessed, or state that a protocol was not prepared.	See methods section; page 2; lines 82-84
	24c	Describe and explain any amendments to information provided at registration or in the protocol.	No amendments were made to the registered protocol.
Support	25	Describe sources of financial or non-financial support for the review, and the role of the funders or sponsors in the review.	See funding section; page 16; line 310
Competing interests	26	Declare any competing interests of review authors.	See conflict of interest section; page 14; line 313
Availability of data, code and other materials	27	Report which of the following are publicly available and where they can be found: template data collection forms; data extracted from included studies; data used for all analyses; analytic code; any other materials used in the review.	No datasets, code, or additional materials were made publicly available. All extracted data are presented in the tables and text of the manuscript.

PRISMA 2020 Checklist

Section and Topic	Item #	Checklist item	Reported (Yes/No)
**TITLE**			
Title	1	Identify the report as a systematic review.	Yes
**BACKGROUND**			
Objectives	2	Provide an explicit statement of the main objective(s) or question(s) the review addresses.	Yes
**METHODS**			
Eligibility criteria	3	Specify the inclusion and exclusion criteria for the review.	Yes
Information sources	4	Specify the information sources (e.g. databases, registers) used to identify studies and the date when each was last searched.	No (exclusion criteria reported in the main text only)
Risk of bias	5	Specify the methods used to assess risk of bias in the included studies.	Yes
Synthesis of results	6	Specify the methods used to present and synthesise results.	No (findings were presented descriptively; no quantitative synthesis or meta-analysis performed).
**RESULTS**			
Included studies	7	Give the total number of included studies and participants and summarise relevant characteristics of studies.	Yes
Synthesis of results	8	Present results for main outcomes, preferably indicating the number of included studies and participants for each. If meta-analysis was done, report the summary estimate and confidence/credible interval. If comparing groups, indicate the direction of the effect (i.e. which group is favoured).	Yes
**DISCUSSION**			
Limitations of evidence	9	Provide a brief summary of the limitations of the evidence included in the review (e.g. study risk of bias, inconsistency and imprecision).	Yes
Interpretation	10	Provide a general interpretation of the results and important implications.	Yes
**OTHER**			
Funding	11	Specify the primary source of funding for the review.	No (reported in the manuscript only)
Registration	12	Provide the register name and registration number.	No (reported in the main text only)

From: Page MJ, McKenzie JE, Bossuyt PM, Boutron I, Hoffmann TC, Mulrow CD, et al. The PRISMA 2020 statement: an updated guideline for reporting systematic reviews. BMJ 2021;372:n71. doi: 10.1136/bmj.n71
